# Four doses of coronavirus disease 2019 vaccination for patients with inborn errors of immunity compared to 3 doses for healthy individuals

**DOI:** 10.1016/j.jacig.2026.100694

**Published:** 2026-03-25

**Authors:** Jeffery C.H. Chan, Samuel M.S. Cheng, Daniel Leung, Xiwei Wang, Manni Wang, Yin Celeste Cheuk, Cyrus Ho, Leo C.H. Tsang, Tsz Chun Kwan, Amos M.T. Lee, Wing Yan Li, Jennifer H.Y. Lam, Kaiyue Zhang, Issan Y.S. Tam, Sau Man Chan, Koon Wing Chan, Malik Peiris, Wenwei Tu, Yu Lung Lau, Jaime S. Rosa Duque

**Affiliations:** a Department of Paediatrics and Adolescent Medicine, The University of Hong Kong, Hong Kong; b School of Public Health, The University of Hong Kong, Hong Kong; c Centre for Immunology & Infection C2i, Hong Kong

**Keywords:** COVID-19, vaccine, inborn errors of immunity

## Abstract

**Background:**

Patients with inborn errors of immunity (IEI) have high risks of severe complications after severe acute respiratory syndrome coronavirus 2 (SARS-CoV-2) infection. Although vaccination is effective in preventing severe coronavirus disease 2019 (COVID-19), boosters are required as the protection provided wanes over time. A greater number of boosters may be required in IEI patients compared to healthy individuals because of their immunodeficient state, but no such data are available.

**Objective:**

We investigated and compared the immunogenicity between 4 doses of COVID-19 vaccination for patients with IEI and 3 doses for healthy individuals.

**Methods:**

In the current study (NCT04800133), either BNT162b2 or CoronaVac was administered as dose 4. Healthy individuals who received 3 doses were included for comparison. Humoral and cellular immunogenicity against wild-type and JN.1 SARS-CoV-2 was assessed between IEI patients and healthy individuals.

**Results:**

IEI patients had lower humoral and cellular immunogenicity than healthy individuals after 3 doses of COVID-19 vaccination. After dose 4, IEI patients obtained immunogenicity similar to that of healthy individuals who received 3 doses. A fourth dose of BNT162b2 significantly enhanced humoral and cellular immunogenicity against wild-type SARS-CoV-2 and humoral responses against JN.1 SARS-CoV-2. In contrast, CoronaVac had minimal effect on humoral responses to wild-type and JN.1 SARS-CoV-2. Six patients experienced breakthrough infections, which did not result in hospitalization or death.

**Conclusion:**

A fourth dose of BNT162b2 was immunogenic and safe for most IEI patients. The fourth dose of vaccination is recommended for IEI patients to improve protection against COVID-19, particularly before travel to areas with high endemic transmission.

In May 2023, the World Health Organization ceased to classify coronavirus disease 2019 (COVID-19) as a public health emergency of international concern; however, immunocompromised patients with inborn errors of immunity (IEI) remain vulnerable.^1^ IEI patients face elevated risks of severe outcomes from COVID-19, including hospitalizations, intensive care unit admissions, deaths, and more reinfections compared to the general population.^2^ Defective type 1 interferon responses due to genetic mutations, as well as neutralizing type 1 interferon autoantibodies, have been shown to be determinants of severe COVID-19.^3^, ^4^, ^5^

BNT162b2 and CoronaVac are among the most widely used COVID-19 vaccines globally, known for their immunogenicity and effectiveness in healthy individuals.^6^, ^7^, ^8^, ^9^ In Hong Kong, population-based observational studies estimated the vaccine effectiveness of BNT162b2 and CoronaVac were approximately 70% to 90%, conferring substantial protection against severe or fatal outcomes.^10^^,^^11^ The vaccine effectiveness of another mRNA vaccine, Moderna, was reported to be 95%.^12^ Our prior research indicates that most IEI patients develop seropositivity and detectable T-cell responses after 3 doses.^13^ The effectiveness of such vaccines was high among IEI patients, as reflected by reductions in the risks of hospitalization and intensive care unit admission by 3.5 times compared to unvaccinated IEI patients.^14^ However, the protection conferred by these vaccines diminishes over time. A review reported the effectiveness against symptomatic disease dropped to 8.9% 9 months after COVID-19 vaccination among healthy individuals.^15^ Because of the immunocompromised states of IEI patients, immunity likely wanes more rapidly, necessitating additional booster doses. Currently, the US Centers for Disease Control and Prevention recommends that moderately or severely immunocompromised individuals receive 4 doses of the COVID-19 vaccination.^16^ However, data on the immunogenicity of dose 4 among IEI patients to support such a recommendation are limited.

Apart from the waning of protection, the emergence of severe acute respiratory syndrome coronavirus 2 (SARS-CoV-2) variants has been another concern. New variants often have higher transmissibility and infectivity as a result of immune escape from host immunity.^17^ JN.1 is a variant of interest designated by the World Health Organization in December 2023.^18^^,^^19^ With high transmissibility, it is believed that JN.1 was responsible for the surge of cases in early 2024.^20^ Because BNT162b2 and CoronaVac are wild-type (WT) COVID-19 vaccines with no antigens from JN.1, it is necessary to investigate the immunogenicity against JN.1 among individuals who received WT vaccines to assess the need for revaccination with JN.1 vaccines, especially in IEI patients.^21^

To understand the safety and immunogenicity of COVID-19 vaccines, we have been conducting a 3-year prospective clinical study (NCT04800133). This interim analysis presents data on the immunogenicity and safety of the fourth dose of COVID-19 vaccines in children and adults with IEI.

## Methods

### Study design

COVID-19 Vaccination in Adolescents and Children (COVAC; registered at ClinicalTrials.gov as NCT04800133 on March 16, 2021) is a clinical study investigating the reactogenicity and immunogenicity of BNT162b2 and CoronaVac among healthy children and adults or those with chronic diseases, as previously described.^7^^,^^13^ The study was approved by the institutional review board of The University of Hong Kong/Hospital Authority Hong Kong West Cluster (HKU/HA HKW IRB, reference UW21-157) and adheres to the Declaration of Helsinki. The use of clinical data and genetic testing results among IEI patients was also approved (HKU/HA HKW IRB, reference UW 08-301).

### Participants

Our analysis consisted of individuals aged 5 years and older with a diagnosis of IEI who had received a minimum of 4 doses of the COVID-19 vaccine. Additionally, a comparative control group of healthy individuals who had received 3 doses of the COVID-19 vaccine was included. For each IEI patient with 1-month post–dose 4 data available, 3 healthy controls were selected on the bases of age, sex, and vaccine type, with an allowable age difference of up to 5 years between the patient and corresponding control. These healthy individuals were participants from the COVAC study.^7^

### Procedures

IEI patients have been followed by the Department of Paediatrics and Adolescent Medicine, The University of Hong Kong. Informed consent was obtained from patients aged 18 years or older. For patients under 18 years old, informed assent and informed consent were obtained from them and their parents or legally acceptable representatives, respectively. A total of 0.3 mL BNT162b2 (equivalent to 30 μg of COVID-19 mRNA vaccine embedded in lipid nanoparticles) or 0.5 mL CoronaVac (600 SU, equivalent to 3 μg of inactivated SARS-CoV-2 virus as antigen) was administered via the intramuscular or intradermal route. An accelerated vaccination regimen was adopted, with dose 4 provided at least 90 days after the preceding dose or previous infection to provide adequate and timely protection. Vaccination was postponed for patients infected with SARS-CoV-2 and was resumed or first administered 28 days later. Blood sampling was performed before dose 3/4 (on the day of vaccination), 1 month after dose 3/4 (13-42 days after dose 3/4), and 6 months after dose 3/4 (126-210 days after dose 3/4). Demographic information was reported by the participants, and their clinical details were extracted from the electronic health records as consented.

### Humoral immunogenicity

Humoral immunogenicity was evaluated by assessing the WT and JN.1 spike receptor–binding domain (S-RBD) IgG levels as well as results from the WT surrogate virus neutralization test (sVNT). S-RBD IgG measured the concentrations of the binding antibody level. sVNT is a functional assay that measures the blocking of S-RBD and human angiotensin-converting enzyme 2 (ACE2) receptor by the vaccine recipient’s circulating antibodies, which correlates with the reference standard plaque reduction neutralization test.^22^

In-house S-RBD IgG enzyme-linked immunosorbent assay (ELISA) was carried out as previously published.^7^^,^^13^^,^^23^ All sera were heat inactivated at 56°C for 30 minutes before testing. In brief, S-RBD IgG ELISA plates were coated overnight with 100 ng per well of purified recombinant S-RBD in phosphate-buffered saline (PBS), followed by the addition of 100 μL Chonblock Blocking/Sample Dilution ELISA buffer (Chondrex, Redmond, Wash). This was incubated at room temperature for 2 hours. The serum was tested at a dilution of 1:100 in Chonblock Blocking/Sample Dilution ELISA buffer, then added to the wells for 2 hours at 37°C. After washing with PBS containing 0.1% Tween 20, horseradish peroxidase–conjugated goat anti-human IgG (1:5,000) (GE Healthcare, Chicago, Ill) was added for 1 hour at 37°C, followed by washing 5 times with PBS containing 0.1% Tween 20. Horseradish peroxidase substrate (Ncm TMB One, New Cell & Molecular Biotech, Suzhou, China) of 100 mL was added for 15 minutes, and the reaction was stopped by 50 mL of 2 mol H_2_SO_4_. The optical density (OD) was analyzed with a Sunrise absorbance microplate reader (Tecan, Männedorf, Switzerland) at 450 nm wavelength. The background OD in PBS-coated control wells with the participant’s serum was subtracted from each OD reading. The limit of detection was set as 0.5. Values at or above an OD_450_ of 0.5 were considered positive, and values below 0.5 were imputed as 0.25 and considered negative.

sVNT was performed according to the manufacturer’s instructions (GenScript, Piscataway, NJ) and as we have described elsewhere.^13^^,^^23^^,^^24^ The sVNT was performed with 10 μL of each serum, positive and negative controls, which were diluted at 1:10 and mixed with an equal volume of horseradish peroxidase conjugated to the WT SARS-CoV-2 S-RBD (6 ng). The mixture was incubated for 30 minutes at 37°C, and then 100 μL of each sample was added to microtiter plate wells coated with ACE2 receptor. This plate was sealed for 15 minutes at 37°C and then washed with wash solution and tapped dry; next, 100 μL of 3,3′,5,5′-tetramethylbenzidine was added and incubated in the dark at room temperature for 15 minutes. This reaction was terminated with 50 μL of Stop Solution, and the absorbance was read at 450 nm in a microplate reader. After confirming the positive and negative controls provided the recommended OD_450_ values, the percentage inhibition of each serum was calculated as [(1 − sample OD value/negative control OD value) × 100%]. Inhibition of at least 30% (the limit of quantification) was regarded as positive, and values below 30% were imputed as 15%. Values between 30% and 90% were regarded as suboptimal.

### Cellular immunogenicity

T-cell responses were assessed for patients with sufficient blood sample volumes and were assessed as we have described elsewhere.^7^^,^^13^ Peripheral blood mononuclear cells were isolated from whole blood by density gradient separation, then frozen in liquid nitrogen until use. Thawed cells were rested for 2 hours in 10% human AB serum supplemented RPMI 1640 medium.

Next, the cells were stimulated with sterile double-distilled water (ddH_2_O) (because the peptides we ordered were dissolved with sterile ddH_2_O) or 1 μg/mL overlapping peptide pools representing the WT SARS-CoV-2 spike (S) proteins (Miltenyi Biotec, Bergisch Gladbach, Germany) or WT reference pool (Miltenyi Biotec) or SARS-CoV-2 S Protein JN.1 (JPT Peptide Technologies, Berlin, Germany) for 16 hours in the presence of 1 μg/mL anti-CD28 and anti-CD49d costimulatory antibodies (clones CD28.2 and 9F10; BioLegend, San Diego, Calif). After 2 hours of stimulation, 10 μg/mL brefeldin A (Sigma, Kawasaki, Japan) was added.

The cells were then washed and subjected to immunostaining using a fixable viability dye (eBioscience, Santa Clara, Calif; 1:60) and antibodies against CD3 (HIT3a, 1:60), CD4 (OKT4, 1:60), CD8 (HIT8a, 1:60), IFN-γ (B27, 1:15), and IL-2 (MQ1-17H12, 1:15) (BioLegend). Data acquisition was carried out by flow cytometry (LSR II; BD Biosciences, Franklin Lakes, NJ) and analyzed by FlowJo v10 software (BD Biosciences).

The antigen-specific IFN-γ^+^ or IL-2^+^ T-cell responses were calculated by subtracting the background (sterile ddH_2_O) data and presented as the percentage of CD4^+^ or CD8^+^ T-cell responses. T-cell response against a single peptide pool was considered positive when the frequency of cytokine-expressing cells was higher than 0.005% and the stimulation index was higher than 2; negative values were imputed as 0.0025%.

### Safety and reactogenicity

Patients reported adverse reactions in an online or paper-based diary for 7 days after vaccination. Unsolicited adverse events were captured up to 28 days after vaccination. Severe adverse events—such as life-threatening complications; unanticipated or prolonged hospitalizations; disabilities; deaths; and breakthrough COVID-19 infection—will be monitored for 3 years after vaccination. The reported safety events were reviewed by the investigators, who determined the possibility of a causal relationship with the study vaccine.

### Statistical analysis

Negative values—that is, those below limit of detection, limit of quantification, or cutoff—were imputed as half of the limit or cutoff and included in the final analyses. Outcome data were compared longitudinally by paired *t* test. Immunogenicity between IEI patients and healthy controls was compared by mixed-effect analysis and the Tukey multiple comparison test. Statistical significance was defined as *P* < .05. Data analysis and graphing were performed by GraphPad Prism v10.0.3 (GraphPad Software, La Jolla, Calif).

## Results

### Study participants

Twenty patients (median age, 17.0 years; 85% male) with IEI were included in the analysis (Table I, and see Table E1 in the Online Repository available at www.jaci-global.org). Six patients experienced breakthrough infection (see Table E2, also in the Online Repository). Of the 20 patients, 15 had 1 month’s post–dose 4 data available. Forty-five healthy individuals (median age, 15.0 years; 89% male) were included as the comparison group.

**Table I:** Patients’ demographic information and clinical characteristics before dose 4

Patient no.	Vaccine brand†	Route‡	Disease category	Age	Sex	Diagnosis	Mutation	History of HSCT	Current medications	ALC (10^9^/L)	CD3 (/μL)	CD19 (/μL)	IgG (mg/dL)	IgA (mg/dL)	IgM (mg/dL)
1	BBBB	MMMM	Humoral	34	M	XLA	XL *BTK:*c.1017-1019delA,p.K296fsX330	No	IVIG	1.05	780	0	1194	<10	<20
2	BBBB	MMMM	Humoral	13	M	XLA	XL *BTK:*c.1111T>C,p.S371P	No	IVIG	1.80	2244	2	1111	<5	<5
3§	BBBB	MMDD	Humoral	13	M	XLA	XL *BTK:*c.1828C>T,p.P566S	No	IVIG	1.70	3310	15	910	<15	<6
4	CCCC	MMMM	Humoral	34	M	XLA	XL *BTK:*c.EX2-EX3del	No	IVIG	2.60			1201	<5	<5
5	CCCC	MMMM	Humoral	14	M	XLA	XL *BTK:*c.1079-1080del,p.T316fs	No	SCIG	2.27	4456	1	1200	<15	<6
6	CCCC	MMMM	Humoral	32	M	XLA	XL *BTK:*c.173C>A,p.S14Y	No	SCIG	1.80	1698	4	1076	182	<20
7	CCCC	MMMM	Humoral	18	M	XLA	XL *BTK:*c.464T>C,p.L111P	No	IVIG	1.50	3552	6	1070	15	6
8§	BBBB	MMMD	Combined	16	F	DN-STAT3	AD *STAT3:*c 2134T>C,p.C712R	No	IVIG	2.25	3928	1435	1700	254	176
9	BBBB	MMMM	Combined	49	M	XLT	XL *WAS:*c.168C>T,p.T45M	No	—	1.10			1230	424	87
10§	BBBB	MMMM	Combined	21	M	AT	—	No	IVIG	0.87	1390	204	1052	<10	<20
11	CCCC	DDDD	Combined	15	M	X-SCID	XL *IL2RG:*c.576C>T,p.Q188∗	Yes	—	2.30	1829	406	1289	180	101
12§	BBBB	MMMM	Dysregulation	18	F	SOCS1	AD *SOCS1:*c.490del,p.A164Pfs∗41	No	IVIG, prednisolone, baricitinib	0.34	257	112	1017	<10	32
13§	BBBB	MMDD	Dysregulation	26	M	XMEN	XL *MAGT1:*c.916del,p.L306fs	No	IVIG	1.42	802	400	958	45	99
14	CCCC	MMMM	Dysregulation	8	M	CINCA	AD *NLRP3:*c. 1711G>C,p.G571R	No	Canakinumab	2.69			1050	138	56
15¶	BBBB	MMDD	Innate	15	M	STAT1-GOF	AD *STAT1:*c.1170G>A,p.M390I	No	—	2.26	1834	538	1490	401	94
16§	CCCC	DDDD	Innate	8	M	CARD9	AR het *CARD9:*c.586A>G,p.K196E and c.1526G>A,p.R509K	No	—	1.91	1915	347	1382	198	156
17	BBBB	MMMM	Phagocytic	14	M	X-CGD	XL *CYBB:*c.483 C>T,p.R157X	No	—	3.14	1506	885	1639	361	188
18	BBBB	MMMM	Phagocytic	50	F	X-CGD	XL *CYBB:*c. 483C>T,p.R157X	No	TAC	0.97			1272	166	127
19	CCCC	MMMM	Phagocytic	11	M	SCN	AD *ELA*2:c.362T>C,p.L121P	No	Filgrastim	2.85			998	64	108
20	CCCC	MMMM	Phagocytic	51	M	SCN	AD *ELA2:*c.362T>C,p.L121P	No	—	1.60			2390	557	147

Immunomodulatory medications, prophylaxis medications, absolute lymphocyte count, CD3 count, CD19 count, IgG, IgA, and IgM levels before dose 4 are listed. *AD,* Autosomal dominant; *ALC,* absolute lymphocyte count; *AR het,* autosomal recessive heterogeneity; *AT,* ataxia telangiectasia; *CARD9,* caspase recruitment domain family member 9 deficiency; *CINCA,* chronic infantile, neurologic, cutaneous, and articular syndrome; *DN-STAT3,* dominant negative STAT3 disease; *HSCT,* hematopoietic stem cell transplantation; *IVIG,* intravenous immunoglobulin; *SCIG,* subcutaneous immunoglobulin; *SCN,* severe congenital neutropenia; *SOCS1,* suppressors of cytokine signaling; *STAT1-GOF,* signal transducer and activator of transcription 1 gain of function mutation; *TAC,* tacrolimus; *X-CGD,* XL chronic granulomatous disease 1; *XL,* X linked; *XLA,* XL agammaglobulinemia; *XLT,* XL thrombocytopenia; *XMEN,* XL immunodeficiency with magnesium defect, Epstein-Barr virus infection, and neoplasia; *X-SCID,* XL severe combined immunodeficiency.

† *B,* BNT162b2; *C,* CoronaVac.

‡ *M,* intramuscular; *D,* intradermal.

§ Individuals with breakthrough infection after dose 4.

¶ Patient 18 (who is mother of patient 17) is carrier of mutation in *CYBB.*

### Suboptimal immunogenicity among IEI patients after 3 doses can be boosted by dose 4

IEI patients had significantly lower geometric mean (GM) levels of S-RBD IgG and sVNT compared to healthy individuals after the third dose, irrespective of the vaccine type received. After administration of dose 4, BNT162b2 recipients obtained antibody levels comparable to those of healthy individuals with 3 doses. For CoronaVac recipients, although the S-RBD IgG and sVNT levels among IEI patients increased, the levels remained lower than those observed in healthy individuals with 3 doses.

**Fig 1:**
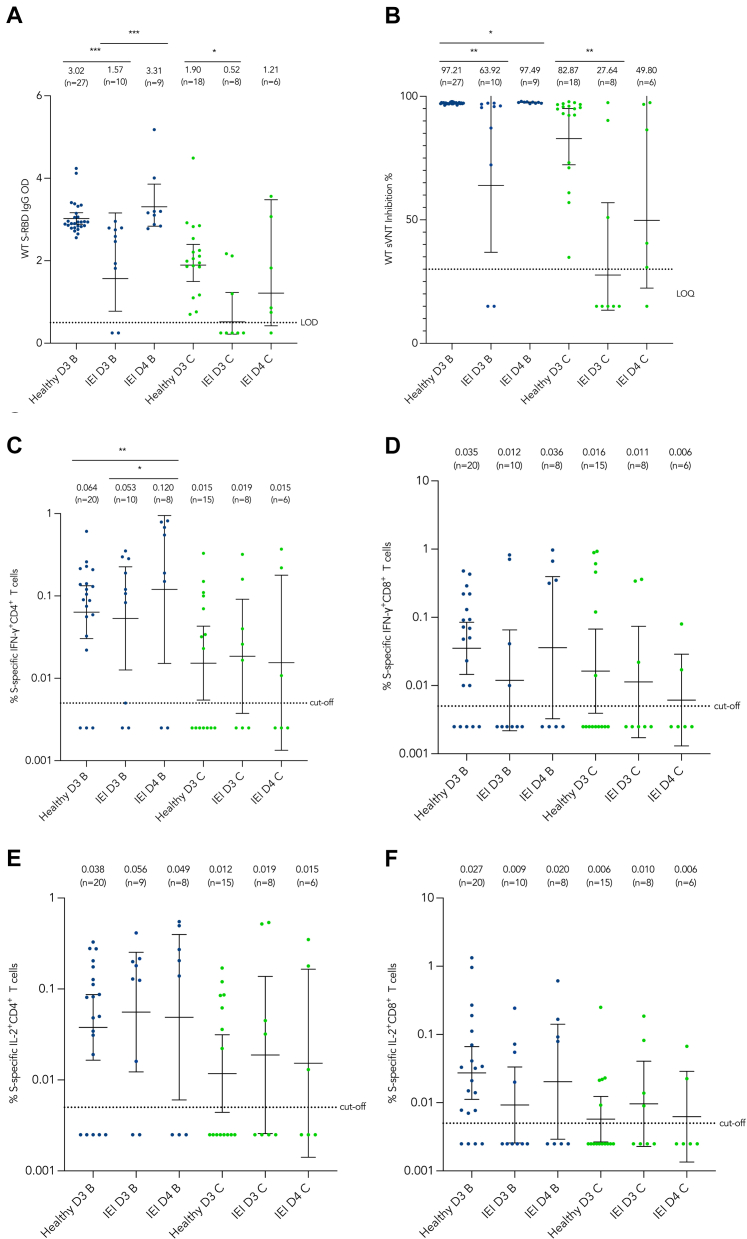
Comparison of humoral immunogenicity among IEI patients receiving dose 3 or 4 with healthy individuals receiving dose 3. WT S-RBD IgG ELISA OD **(A)** and WT sVNT inhibition percentage level **(B)** at 1 month after dose 3/4 were compared between IEI patients receiving dose 4, IEI patients receiving dose 3, and healthy individuals receiving dose 3. GMs with 95% confidence intervals are indicated by *center lines.* GMs with number of samples are shown above each column. Limit of detection (0.5) and limit of quantification (30%) are indicated by *dotted lines. B,* BNT162b2; *C,* CoronaVac; *D3,* individuals receiving dose 3; *D4,* individuals receiving dose 4; *IEI,* inborn errors of immunity; *LOD,* limit of detection; *LOQ,* limit of quantification. ∗*P* < .05, ∗∗*P* < .01, ∗∗∗*P* < .001.

Regarding the cellular immunogenicity among individuals receiving BNT162b2, the S-specific IFN-γ^+^CD4^+^, IFN-γ^+^CD8^+^, and IL-2^+^CD8^+^ T-cell responses among IEI patients were lower than those of healthy individuals after 3 doses (Fig 1, *C-F*). After dose 4 of BNT162b2, the IFN-γ^+^CD4^+^ T-cell response was significantly higher than that of healthy individuals and IEI patients after 3 doses. For CoronaVac recipients, T-cell responses showed no substantial differences between healthy individuals and IEI patients with 3 or 4 doses.

### BNT162b2 provided higher humoral immunogenicity than CoronaVac after 4 doses

Before dose 4, a total of 4 patients (all CoronaVac recipients) were negative for both S-RBD IgG and sVNT. Two patients had humoral deficiencies, while the other two had phagocytic and combined deficiencies, respectively (see Table E3 in the Online Repository available at www.jaci-global.org). After dose 4, there were notable increases in the GM S-RBD IgG and sVNT levels in both the BNT162b2 and CoronaVac groups.

**Fig 2:**
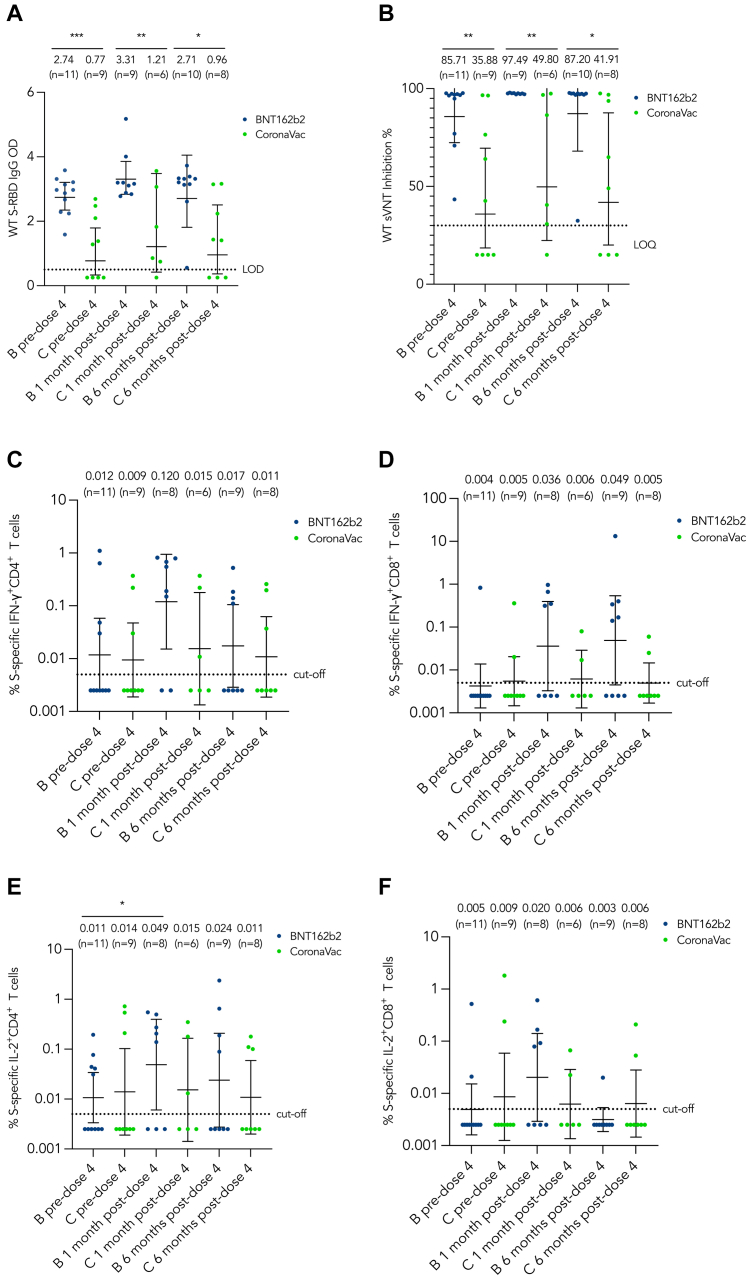
Longitudinal analysis of humoral and cellular immunogenicity against WT SARS-CoV-2. WT S-RBD IgG ELISA OD **(A),** WT sVNT inhibition percentage level **(B),** and WT S-specific IFN-γ^+^IL-2^+^CD4^+^CD8^+^ T cells **(C-F)** were shown longitudinally. GMs with 95% confidence intervals are indicated by *center lines.* GMs with number of samples is shown above each column. Limit of detection (0.5), limit of quantification (30%), or cutoffs (0.005) are indicated by *dotted lines. Blue* and *green dots* represent data from patients receiving BNT162b2 and CoronaVac, respectively. *B,* BNT162b2; *C,* CoronaVac; *LOD,* limit of detection; *LOQ,* limit of quantification.

Of the 4 patients who were seronegative before dose 4, a single individual, with a humoral defect, remained negative for S-RBD IgG and sVNT at 1 month after dose 4. At 6 months after dose 4, the antibody levels dropped and were comparable to pre–dose 4 levels in both vaccine groups (Fig 2, *A* and *B*). Three patients were negative for S-RBD IgG and sVNT at 6 months after dose 4, all of whom were seronegative at the pre–dose 4 time point (Table E3).

Throughout the 3 timepoints, S-RBD IgG and sVNT levels were significantly lower among CoronaVac recipients than BNT162b2 recipients (Fig 2, *A* and *B*). The disparity between the groups that received the different vaccine types became smaller at 6 months after dose 4 compared to 1 month after dose 4 but still remained significant.

### BNT162b2 but not CoronaVac boosted cellular immunogenicity

At the pre–dose 4 time point, most patients had T-cell responses lower than the cutoff (Fig 2, *C-F*). For patients receiving BNT162b2, an increase in S-specific IFN-γ^+^CD4^+^, IFN-γ^+^CD8^+^, IL-2^+^CD4^+^, and IL-2^+^CD8^+^ T cells was observed at 1 month after dose 4. At 6 months after dose 4, only GM IFN-γ^+^CD8^+^ T cells persisted at a high level, whereas GM of IFN-γ^+^CD4^+^, IL-2^+^CD4^+^, and IL-2^+^CD8^+^ cells dropped to levels comparable to the pre–dose 4 time point. The change in T-cell responses among CoronaVac recipients was minimal across the 3 time points. The T-cell responses among BNT162b2 recipients were generally better than those of CoronaVac recipients at 1 month after dose 4.

### Immunogenicity against JN.1

**Fig 3:**
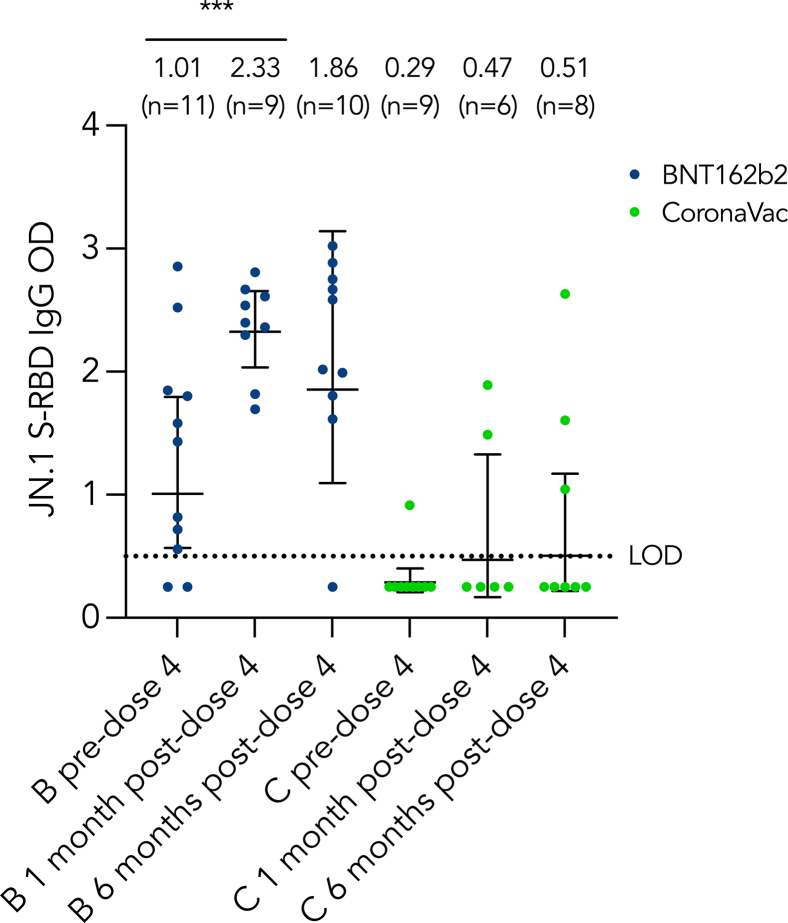
Longitudinal analysis of humoral and cellular immunogenicity against JN.1 SARS-CoV-2. JN.1 S-RBD IgG ELISA OD **(A)** and JN.1 S-specific IFN-γ^+^IL-2^+^CD4^+^CD8^+^ T cells **(B-E)** are shown longitudinally. GMs with 95% confidence intervals are indicated by *center lines.* GMs with number of samples are shown above each column. Limit of detection (0.5), limit of quantification (30%), or cutoffs (0.005) are indicated by *dotted lines. C,* CoronaVac; *LOD,* limit of detection; *LOQ,* limit of quantification. ∗∗∗*P* < .001.

BNT162b2 recipients generally had higher JN.1-specific S-RBD IgG levels than CoronaVac recipients before and after dose 4 (Fig 3). For BNT162b2 recipients, the S-RBD IgG level increased significantly by 2-fold at 1 month after dose 4 compared to before dose 4. The level dropped at 6 months after dose 4, but the change did not reach any significance.

### Breakthrough infection was mild among IEI patients after 4 doses

Six patients (30%) experienced breakthrough COVID-19 after the fourth dose, among whom 2, 2, 1, and 1 patients had combined, dysregulation, humoral, and innate defects, respectively (Table E2). No hospitalization or death was reported. The 5 patients who received BNT162b2 and experienced breakthrough infections after dose 4 had humoral and cellular immunogenicity similar to those of patients without breakthrough infections at 1 month after dose 4 (see Fig E1 in the Online Repository available at www.jaci-global.org).

### Adverse reactions were common but mild

**Fig 4:**
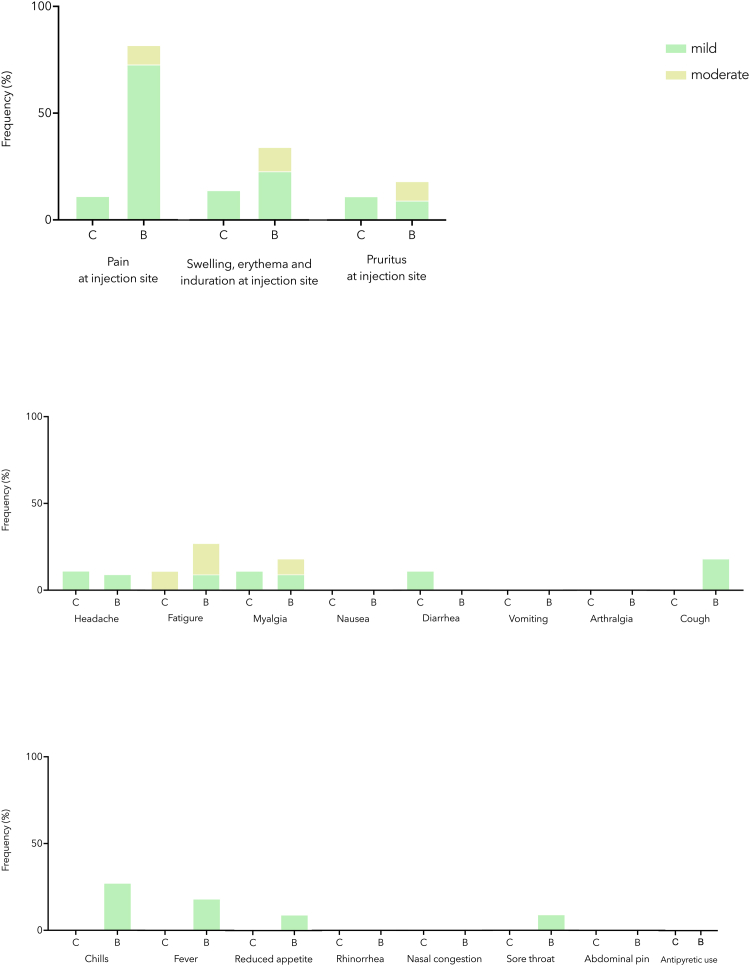
Adverse reactions and antipyretic receipt reported within 7 days after dose 4. Adverse reactions were reported as mild or moderate. *B,* BNT162b2; *C,* CoronaVac.

Only mild and moderate adverse reactions were reported by patients after dose 4 (Fig 4). For BNT162b2 recipients, the 3 most reported adverse reactions were pain at the injection site (82%), fatigue (27%), and swelling around the injection site (27%). For CoronaVac recipients, fatigue (11%), pain at the injection site (11%), swelling at the injection site (11%), headache (11%), myalgia (11%), diarrhea (11%), and pruritus at the injection site (11%) were reported.

## Discussion

This study underscores the suboptimal immunogenicity of three COVID-19 vaccine doses in IEI patients compared to healthy individuals. Administering a fourth BNT162b2 dose to this group of patients elicited humoral and cellular immune responses similar to those of healthy individuals after 3 doses, whereas the immunogenicity induced by CoronaVac was notably lower. Furthermore, the fourth BNT162b2 dose significantly enhanced humoral responses against the JN.1 SARS-CoV-2 variant. Although breakthrough infections occurred in some patients, there were no hospitalizations or deaths.

The impact of COVID-19 has notably diminished in recent months and is anticipated to continue decreasing in the foreseeable future.^25^ Nevertheless, IEI patients and individuals with immunodeficient states remain at a heightened risk of severe COVID-19 if infected. Regular vaccination against SARS-CoV-2 is recommended in this vulnerable group. The current recommendation for individuals who are moderately or severely immunocompromised is a 4-dose series, and a fifth dose may be required if individuals received their last dose before 2024.^26^

Our study underscores the disparity in antibody levels between IEI patients and healthy individuals after 3 doses of COVID-19 vaccination. Additional booster doses are crucial to provide adequate protection. With the fourth dose of BNT162b2, IEI patients gained comparable antibody levels to healthy individuals receiving 3 doses. However, IEI patients receiving the fourth CoronaVac exhibited lower antibody responses than healthy individuals receiving only 3 doses. Further doses of mRNA-based rather than inactivated vaccines may be considered for this patient population.

Antibodies play a crucial role as the primary defense mechanism against SARS-CoV-2 that aid in the prevention of breakthrough infections.^6^^,^^27^^,^^28^ In our study, although most patients were already seropositive before dose 4, we observed significant increases in WT S-RBD IgG positivity and GM sVNT by 13% and 40%, respectively, at 1 month after dose 4. Similar findings have been reported by other research groups. For instance, a study conducted in Belgium demonstrated a 40% increase in median antibody levels among IEI patients after dose 4.^29^ No statistically significant waning was detected in our study, which could be attributed to insufficient time for waning to occur. Further follow-up investigations are needed to monitor the durability of the additional humoral responses provided by the fourth dose of the COVID-19 vaccines to optimize timing for further doses if needed.

T cells play a crucial role as key regulators of the immune system’s response to COVID-19.^30^ They are essential for virus clearance, as they can recognize and form immune memory of antigens, providing long-term protection.^6^^,^^31^, ^32^, ^33^ In our study, BNT162b2 boosted cellular responses while CoronaVac did not at 1 month after dose 4. In terms of the durability of cellular responses induced by BNT162b2, T-cell responses of most patients dropped back to pre–dose 4 levels at 6 months after dose 4. The cellular responses were found to be stable and not further increased after completion of initial doses in another study.^34^ It is suggested that after the full vaccination regimen, cellular immunity is typically established, and additional booster doses may not significantly alter existing immunity levels.

In this study, BNT162b2 recipients generally had higher S-specific T-cell responses compared to those receiving CoronaVac after the fourth dose. A study conducted in Singapore demonstrated that although T-cell responses against S protein were lower among CoronaVac recipients, the T-cell responses against multiple viral proteins were similar between CoronaVac and BNT162b2 recipients.^35^ The variance in S-specific T-cell responses between CoronaVac and BNT162b2 recipients could be attributed to the compositions of the vaccines. BNT162b2, an mRNA vaccine, encodes the S protein of SARS-CoV-2, while CoronaVac, an inactivated vaccine, comprises not only the S protein but also other viral proteins such as the membrane and nucleocapsid proteins.^36^ The S protein is crucial because it facilitates cell recognition and membrane fusion, making it a key target for immune responses.^37^ While CoronaVac triggers T-cell responses against multiple viral proteins, BNT162b2 primarily focuses on inducing a response against the S protein.^7^

In terms of the humoral responses toward JN.1, BNT162b2 significantly boosted JN.1 S-RBD IgG levels, while CoronaVac did not. The JN.1 S-RBD IgG positivity and GM value increased by 18% and 130%, respectively, among BNT162b2 recipients after dose 4. Although the antibody levels dropped at 6 months after dose 4, the reduction was not statistically significant. Given the limited immunogenicity against the JN.1 variant provided by dose 4 of either BNT162b2 or CoronaVac, vaccination with the JN.1 lineage COVID-19 mRNA vaccines should be considered for IEI patients. Indeed, the recommendation for vaccination with the JN.1 lineage was proposed by the World Health Organization in April 2024.^38^ Studies showed that the JN.1 vaccines are immunogenic among healthy individuals, but data on the immunogenicity of JN.1 vaccination among IEI patients have been scarce.^39^

Immunoglobulin replacement therapy was administered to 11 patients in the study, most of whom had humoral defects. Immunoglobulin replacement therapy is beneficial for individuals with impaired antibody production in reducing the risk and severity of infections.^40^^,^^41^ Its protective effects are mediated through several mechanisms, including activation of the complement system, modulation of Fc receptor expression and function, and neutralizing T-cell superantigens.^42^ Recent studies have shown most immunoglobulin products manufactured after 2020 contain antibodies against SARS-CoV-2. Notably, their ability to inhibit ACE2-binding activity increases with later expiration dates but diminishes against the more recent viral variants.^43^ The acquisition of anti-S antibodies from immunoglobulin replacement therapy may therefore offer additional protection against severe COVID-19 in patients with antibody deficiencies.^6^^,^^44^ This occurs likely via the activation of S-specific antibody-dependent cellular cytotoxicity, which enhances Fc-mediated antiviral functions and provides early immune protection against COVID-19.^45^^,^^46^

In our study, patients with humoral defects exhibited lower cellular immune responses. This may reflect impaired or absent interactions between B cells and T cells, which are required for effective adaptive immunity. A recent review on T-cell function in individuals with primary antibody deficiencies highlighted several abnormalities, including reduced T follicular helper cells, markedly decreased T_H_17, regulatory T, and memory T-cell subsets, and altered T-cell receptor repertoire.^47^ This is consistent with our findings that patients with humoral defects had diminished cellular responses to COVID-19 vaccination.

This study had several strengths and limitations. In addition to WT humoral and cellular responses, we also studied responses toward the JN.1 variant. We were able to track patients’ responses from before dose 4 to 6 months after dose 4. However, because IEI is a rare disease, the number of patients was small; we can only present the results, but we note that it is not possible to provide definite conclusions on the cellular immunogenicity of these vaccines against COVID-19. In the future, pooling data and publications, perhaps by collaborative efforts and meta-analysis, will enhance analysis accuracy and permit us to study single IEI with less heterogeneity. Because the breakthrough infections were self-reported by the patients, the study results did not account for asymptomatic infections. It was not possible to include a detailed history for every patient because some also received clinical care outside what we provided and beyond our electronic health record system, such as those with acute illnesses requiring urgent attention by local primary providers or those often traveling abroad.

In conclusion, 3 doses of COVID-19 vaccine may not provide adequate immunogenicity to IEI patients. By receiving a fourth dose of BNT162b2, patients obtained higher immunogenicity that was comparable to healthy individuals. However, on the basis of the observed suboptimal responses to CoronaVac, IEI patients who received CoronaVac should consider COVID-19 mRNA-based vaccination containing JN.1 or other emerging variants of concern. Further studies are required to track and understand the waning of humoral and cellular responses after the fourth dose to optimize the timing of more boosters, if indicated.

**Key messages**

• Four doses of BNT162b2 induced similar immunogenicity for patients with IEI as 3 doses for healthy individuals.
• It is recommended that those with IEI who received CoronaVac should receive an mRNA-based vaccine because of suboptimal responses.

## Disclosure statement

Supported by research grants (COVID19F02, COVID19F10, and COVID19F12) from the Hong Kong SAR Government, which was not involved in the study design, performance, interpretation, or publication of the project. Genetic diagnosis of IEI among the patients was funded by The Society for the Relief of Disabled Children, Hong Kong.

Disclosure of potential conflict of interest: The authors declare that they have no relevant conflicts of interest.
