# Supplementary material for: Four doses of coronavirus disease 2019 vaccination for patients with inborn errors of immunity compared to 3 doses for healthy individuals
Source: J Allergy Clin Immunol Glob. 2026 Mar 25;5(4):100694. doi: 10.1016/j.jacig.2026.100694 (PMC13098406; doi:10.1016/j.jacig.2026.100694)
Supplement: Supplementary Fig and Tables [file mmc1.docx]

**Supplementary Table 1. Brief history of each patient.**

| Patient No. | Age | Sex | Diagnosis | Mutation | History |
| --- | --- | --- | --- | --- | --- |
| 1 | 34 | M | XLA | XL *BTK*:c.1017-1019delA,p.K296fsX330 | He had experienced recurrent respiratory tract infections from late infancy until he commenced intravenous immunoglobulin. High-resolution computed tomography (HRCT) was done at 32 years old, which revealed patchy ground‑glass opacification at the lingular segment of the left upper lobe and subcentimeter ground‑glass nodules at the right lower lobe. With regular IVIG, he was asymptomatic. |
| 2 | 13 | M | XLA | XL *BTK*:c.1111T>C,p.S371P | He had experienced recurrent upper respiratory infections, pneumonia, and gastroenteritis since 7 years old. He had an episode of otitis media at 11 years old and pyogenic arthritis requiring arthrotomy in the left hip at 12 years old. He was diagnosed with hypogammaglobulinemia at 12 years old. He had mild bronchiectasis in the left lower lobe with scattered fibrosis. With regular IVIG, he maintained a stable condition. |
| 3* | 13 | M | XLA | XL *BTK*:c.1828C>T,p.P566S | He had recurrent pneumonia starting at 5 years old and received an XLA diagnosis at 10 years old. Regular IVIG was commenced after diagnosis. He had bronchiectasis with fixed obstructive lung disease. |
| 4 | 34 | M | XLA | XL *BTK*:c.EX2-EX3del | He had experienced recurrent otitis media before he commenced regular IVIG. He had severe sinusitis at 23 years old and underwent functional endoscopic sinus surgery for right sinusitis at 30 years old. He was under regular IVIG. |
| 5 | 14 | M | XLA | XL *BTK*:c.1079-1080del,p.T316fs | He had experienced recurrent sinopulmonary infections (pneumonia, otitis media, bronchitis) and extended‑spectrum beta‑lactamase‑producing *E. coli* septicemia. He had bronchiectasis with impaired lung function requiring lung rehabilitation. He had received IVIG since 7 years old and switched to subcutaneous immunoglobulin (SCIG) at 16 years old. |
| 6 | 32 | M | XLA | XL *BTK*:c.173C>A,p.S14Y | He was a hepatitis B carrier with recurrent pneumonia in adolescence and was diagnosed with XLA at 16 years old. He responded well to SCIG with no recent infections. |
| 7 | 18 | M | XLA | XL *BTK*:c.464T>C,p.L111P | He presented at 2 years old with recurrent infections. Regular IVIG had been administered since diagnosis. He did not have significant breakthrough infections or bronchiectasis. |
| 8* | 16 | F | DN-STAT3 | AD *STAT3*:c 2134T>C,p.C712R | She had recurrent skin abscesses and pneumonia, resulting in pneumatocele formation. She had a static cavitation lesion in the right upper lobe of the lung. She had hypothyroidism requiring thyroxine, anorexia nervosa, and acne excoriée. She received monthly IVIG. |
| 9 | 49 | M | XLT | XL *WAS*:c.168C>T,p.T45M | He initially presented with petechiae at 1 month old. He had bruises when he began walking. He had several nosebleeds and gum infections in childhood. Splenectomy was performed at 8 years old, and an accessory splenectomy was performed at 20 years old. He experienced a major gastrointestinal bleeding at 12 years old. Bone marrow examination was done at 25 years old, revealing thrombocytopenia with increased megakaryocytopoiesis. |
| 10* | 21 | M | AT | / | He was diagnosed at 4 years old, initially presenting as a wobbling gait. He did not have frequent respiratory infections in early life, but after 6 years old, the frequency increased to 5–6 episodes per year, each lasting 2 weeks. He had bronchiectasis with suspected restrictive lung disease. |
| 11 | 15 | M | X-SCID | XL *IL2RG*:c.576C>T,p.Q188* | He had T–B+NK– SCID with disseminated BCG infection following vaccination. He received a bone marrow transplant at 9 months old with a 10/10 matched unrelated donor. He underwent splenectomy for multiple splenic BCG abscesses at 11 months old. After transplant, he had a viral wart excised at 15 years old and had small warty lesions on the left palm and foot with multiple episodes of left posterior thigh spontaneous discharge since 12 years old. |
| 12* | 18 | F | SOCS1 | AD *SOCS1*:c.490del,p.A164Pfs*41 | She initially presented at 10 years old with pyrexia of unknown origin and lymphadenopathy. She had massive splenomegaly since 16 years old. She had multisystem autoimmunity (cytopenia, presumed autoimmune hepatitis), atopic dermatitis, combined immune deficiency (T and NK cell lymphopenia, impaired B memory switch, hypogammaglobulinemia), and lymphoproliferation (splenomegaly and lymphadenopathy). |
| 13* | 26 | M | XMEN | XL *MAGT1*:c.916del,p.L306fs | He was diagnosed with XMEN at 5 years old during workup for autoimmune hepatitis, hypogammaglobulinemia, and recurrent sinopulmonary infections. He had deranged liver function since 3 years old without chronic liver disease. He had a relapse of immune thrombocytopenia at 24 years old. |
| 14 | 8 | M | CINCA | AD *NLRP3*:c. 1711G>C,p.G571R | He initially presented with a generalized rash on the first day of life and fever since 1 week old. MRI showed ventriculomegaly with white matter thinning and enlarged CSF spaces. He had significant allergic rhinitis and conjunctivitis with regular inhaled corticosteroid use. He was on canakinumab. |
| 15# | 15 | M | STAT1 GOF | AD *STAT1*:c.1170G>A,p.M390I | He had had recurrent febrile episodes caused by tonsillitis, oral ulcers, and diarrhoea since childhood. He had fungal nail infection since 3 years old, beginning on the left fifth toe and later involving four toes. He was diagnosed with chronic mucocutaneous candidiasis. |
| 16* | 8 | M | CARD9 | AR het *CARD9*:c.586A>G,p.K196E and c.1526G>A,p.R509K | He presented with headache and a left upper limb seizure. MRI revealed a right parietal focal enhancing mass with vasogenic oedema and midline shift. Histopathology and sequencing confirmed phaeohyphomycosis. At 1 year follow‑up, he had near-complete neurological and radiological recovery. |
| 17 | 14 | M | X-CGD | XL *CYBB*:c.483 C>T,p.R157X | He initially presented with diarrhoea and dehydration on day 22 of life, and pustules over the left thigh. Perianal abscesses formed at 14 months old and required incision and drainage. He had *Salmonella* septicemia at 2 years old. He had multiple small fistula openings without discharge and a left submandibular abscess. He had an absent oxidative burst response. |
| 18 | 50 | F | X-CGD | XL *CYBB*:c. 483C>T,p.R157X | She was the mother of patient #17. She had a history of lupus rash and stage 1 cervical cancer, treated with chemoradiotherapy at 48 years old. No further immunological history was retrievable. |
| 19 | 11 | M | SCN | AD *ELA*2:c.362T>C,p.L121P | He had left-shifted granulopoiesis with relative eosinophilia at 11 years old and had no evidence of dysplasia. Bone marrow exam showed no myelodysplasia. His condition was controlled with G‑CSF. |
| 20 | 51 | M | SCN | AD *ELA2*:c.362T>C,p.L121P | He had recurrent skin infections with no response to G‑CSF. He had ischemic bowel requiring right hemicolectomy at 17 years old and had mild chronic gastritis at 50 years old. |

A brief history of each patient included in this study is provided below. The history of some of the patients was reported previously. Patient #1-3,5-7 were included in a case series describing the outcomes of X-linked agammaglobulinemia patients [1]. Patient #14 was included in a case series to report the effect of canakinumab on cryopyrin-associated periodic syndrome [2]. Patient #16 was reported as a case report [3]. Patient #17 was included in a case series for phenomic analysis [4].

Nonetheless, some of the patients did not receive medical care in the public setting, and we could not retrieve their detailed history from the electronic health record.

1. Shillitoe B, Duque JSR, Lai SHY, Lau TM, Chan JCH, Bourne H, et al. Outcomes of X-Linked Agammaglobulinaemia Patients. J Clin Immunol. 2024;45(1):40.

2. Shu Z, Zhang Y, Han T, Li Y, Piao Y, Sun F, et al. The genetic and clinical characteristics and effects of Canakinumab on cryopyrin-associated periodic syndrome: a large pediatric cohort study from China. Front Immunol. 2023;14:1267933.

3. Lai SHY, Duque JSR, Chung BH-Y, Chung TW-H, Leung D, Ho RS-L, et al. Invasive cerebral phaeohyphomycosis in a Chinese boy with CARD9 deficiency and showing unique radiological features, managed with surgical excision and antifungal treatment. International Journal of Infectious Diseases. 2021;107:59-61.

4. Chiu TL, Leung D, Chan KW, Yeung HM, Wong CY, Mao H, et al. Phenomic Analysis of Chronic Granulomatous Disease Reveals More Severe Integumentary Infections in X-Linked Compared With Autosomal Recessive Chronic Granulomatous Disease. Front Immunol. 2021;12:803763.

**Supplementary Table 2. Details of vaccination and breakthrough infection.**

| Patient Number | Vaccine type | Date of vaccination | Date of breakthrough infection | Number of days |
| --- | --- | --- | --- | --- |
| 1 | B | 04/09/2022 | N/A | N/A |
| 2 | B | 07/08/2022 | N/A | N/A |
| 3 | B | 22/05/2022 | 01/09/2022 | 102 days |
| 4 | C | 26/03/2022 | N/A | N/A |
| 5 | C | 06/08/2022 | N/A | N/A |
| 6 | C | 12/03/2022 | N/A | N/A |
| 7 | C | 12/03/2022 | N/A | N/A |
| 8 | B | 10/07/2022 | 02/01/2023 | 176 days |
| 9 | B | 13/03/2022 | N/A | N/A |
| 10 | B | 10/07/2022 | 12/12/2022 | 155 days |
| 11 | C | 03/09/2022 | N/A | N/A |
| 12 | B | 09/07/2022 | 20/11/2022 | 134 days |
| 13 | B | 07/08/2022 | 22/10/2022 | 76 days |
| 14 | C | 03/09/2022 | N/A | N/A |
| 15 | B | 12/06/2022 | N/A | N/A |
| 16 | C | 06/08/2022 | 03/09/2022 | 28 days |
| 17 | B | 04/09/2022 | N/A | N/A |
| 18 | B | 04/09/2022 | N/A | N/A |
| 19 | C | 06/08/2022 | N/A | N/A |
| 20 | C | 06/08/2022 | N/A | N/A |

B, BNT162b2; C, CoronaVac; N/A, not applicable.

**Supplementary Table 3. Humoral and cellular immunogenicity against wild type SARS-CoV-2 by disease category.**

|  | Healthy control | All IEI patients | Combined | Dysregulation | Humoral | Innate | Phagocytic |
| --- | --- | --- | --- | --- | --- | --- | --- |
| **S-RBD IgG seropositive (%)** | | | | | | | |
| 1-month post-dose 3 | 45/45 (100%) | 11/18 (61%) | 1/3 (33%) | 3/3 (100%) | 3/7 (43%) | 1/1 (100%) | 3/4 (100%) |
| Pre-dose 4 | N/A | 16/20 (80%) | 3/4 (75%) | 3/3 (100%) | 5/7 (71%) | 2/2 (100%) | 3/4 (75%) |
| 1 month post-dose 4 | N/A | 14/15 (93%) | 3/3 (100%) | 3/3 (100%) | 5/6 (83%) | 1/1 (100%) | 2/2 (100%) |
| 6 months post-dose 4 | N/A | 15/18 (83%) | 3/4 (75%) | 3/3 (100%) | 4/6 (67%) | 1/1 (100%) | 4/4 (100%) |
| **Geometric mean sVNT % inhibition level (95% CI)** | | | | | | | |
| 1 month post-dose 3 | 91.2 (86.2-96.5) | 44.0 (28.2-68.8) | 27.8 (1.9-398.1) | 94.1 (85.9-103.2) | 29.2 (13.3-63.9) | 87.2 | 61.0 (13.8-269.8) |
| Pre-dose 4 | N/A | 57.9 (41.0-81.8) | 61.0 (13.8-269.9) | 67.8 (24.2-189.8) | 52.5 (23.6-116.7) | 64.8 (0.4-10476) | 55.0 (13.4-225.8) |
| 1 month post-dose 4 | N/A | 74.5 (54.4-102.1) | 97.4 (96.6-98.2) | 97.2 (96.1-98.3) | 57.8 (24.9-133.9) | 97.2 | 62.9 (0.2-16527) |
| 6 months post-dose 4 | N/A | 63.0 (43.9-90.3) | 61.1 (13.8-270.6) | 85.1 (47.6-152.4) | 38.7 (15.9-94.2) | 97.5 | 96.2 (93.5-99.0) |
| **Geometric mean IFN-γ^+^ CD4^+^ (95% CI)** | | | | | | | |
| 1 month post-dose 3 | 0.0345 (0.0184-0.0646) | 0.0334 (0.0124-0.0896) | 0.1961 (0.0307-1.255) | 0.0123 (1.29*10^-5-11.76) | 0.0577 (0.0117-0.2839) | 0.005 | 0.0115 (0.0006-0.219) |
| Pre-dose 4 | N/A | 0.0083 (0.0032-0.0215) | 0.0047 (0.0006-0.0336) | 0.0025 (0.0025-0.0025) | 0.0225 (0.0013-0.4008) | 0.0025 (0.0025-0.0025) | 0.1223 (0.0017-8.679) |
| 1 month post-dose 4 | N/A | 0.0499 (0.0115-0.2171) | 0.0686 (0-101.5) | 0.1027 (0-307.9) | 0.0076 (0.0008-0.0696) | 0.817 | 0.2853 (0.0105-7.757) |
| 6 months post-dose 4 | N/A | 0.0139 (0.0045-0.0424) | 0.0064 (0.0003-0.1307) | 0.0025 (0.0025-0.0025) | 0.0535 (0.0043-0.6726) | 0.037 | 0.0105 (0-4.986) |
| **Geometric mean IFN-γ^+^ CD8^+^ (95% CI)** | | | | | | | |
| 1 month post-dose 3 | 0.0253 (0.0119-0.0539) | 0.0116 (0.0038-0.0357) | 0.0204 (3.78*10^-5-11.02) | 0.0025 (0.0025-0.0025) | 0.0356 (0.0028-0.4549) | 0.041 | 0.0025 (0.0025-0.0025) |
| Pre-dose 4 | N/A | 0.0048 (0.0022-0.0105) | 0.0025 (0.0025-0.0025) | 0.0025 (0.0025-0.0025) | 0.0117 (0.0010-0.1339) | 0.0071 (0-3863) | 0.0025 (0.0025-0.0025) |
| 1 month post-dose 4 | N/A | 0.0168 (0.0040-0.0701) | 0.0126 (0-12.97) | 0.0182 (0-93.98) | 0.0099 (0.0007-0.1443) | 0.665 | 0.0141 (0-51628189) |
| 6 months post-dose 4 | N/A | 0.0167 (0.0043-0.0649) | 0.0189 (0.0004-0.9183) | 0.0102 (0-4.29) | 0.0357 (0.0009-1.445) | 0.0025 | 0.0096 (0-3.076) |
| **Geometric mean IL-2^+^ CD4^+^ (95% CI)** | | | | | | | |
| 1 month post-dose 3 | 0.0229 (0.0122-0.0432) | 0.0334 (0.0109-0.1022) | 0.2053 (0.0257-1.638) | 0.0025 (0.0025-0.0025) | 0.0399 (0.0057-0.2770) | 0.016 | 0.0276 (0.0003-2.411) |
| Pre-dose 4 | N/A | 0.0120 (0.0045-0.0322) | 0.0312 (0.0017-0.5843) | 0.0078 (0-1.069) | 0.0150 (0.0016-0.1394) | 0.0367 (0-2.5*10^13) | 0.0025 (0.0025-0.0025) |
| 1 month post-dose 4 | N/A | 0.0297 (0.0075-0.1180) | 0.0696 (0-92.81) | 0.0151 (0-34.43) | 0.0084 (0.0008-0.0915) | 0.139 | 0.2499 (0.0036-17.38) |
| 6 months post-dose 4 | N/A | 0.0165 (0.0047-0.0577) | 0.0853 (0.0010-7.374) | 0.0025 (0.0025-0.0025) | 0.0119 (0.0009-0.1594) | 0.178 | 0.0106 (0-5.228) |
| **Geometric mean IL-2^+^ CD8^+^ (95% CI)** | | | | | | | |
| 1 month post-dose 3 | 0.0140 (0.0074-0.0264) | 0.0094 (0.0040-0.0221) | 0.0491 (0.0071-0.3404) | 0.0025 (0.0025-0.0025) | 0.0176 (0.0027-0.1177) | 0.0025 | 0.0034 (0.0012-0.0095) |
| Pre-dose 4 | N/A | 0.0063 (0.0024-0.0166) | 0.0078 (0.0002-0.2943) | 0.0025 (0.0025-0.0025) | 0.0034 (0.0016-0.0071) | 0.0673 (0-9.9*1-^16) | 0.0095 (0.0001-0.6633) |
| 1 month post-dose 4 | N/A | 0.0122 (0.0038-0.0393) | 0.0494 (0-49) | 0.0025 (0.0025-0.0025) | 0.0080 (0.0010-0.0631) | 0.167 | 0.0129 (0-1.5*10^7) |
| 6 months post-dose 4 | N/A | 0.0044 (0.0022-0.0087) | 0.0090 (0.0008-0.1037) | 0.0025 (0.0025-0.0025) | 0.0052 (0.0008-0.0349) | 0.0025 | 0.0025 (0.0025-0.0025) |

S-RBD IgG seropositivity was expressed as the number of participants tested positive over the total number of participants at the specified time point. The 1 month post-dose 3 results in healthy and patients with IEI were included as reference. There was no pre- and post-dose 4 data available for healthy individuals as they did not receive the fourth doses. IEI, inborn errors of immunity; S-RBD, spike receptor-binding domain; sVNT, surrogate virus neutralization test; CI, confidence interval; IFN-γ, interferon gamma; IL-2 interleukin 2; N/A, not applicable.


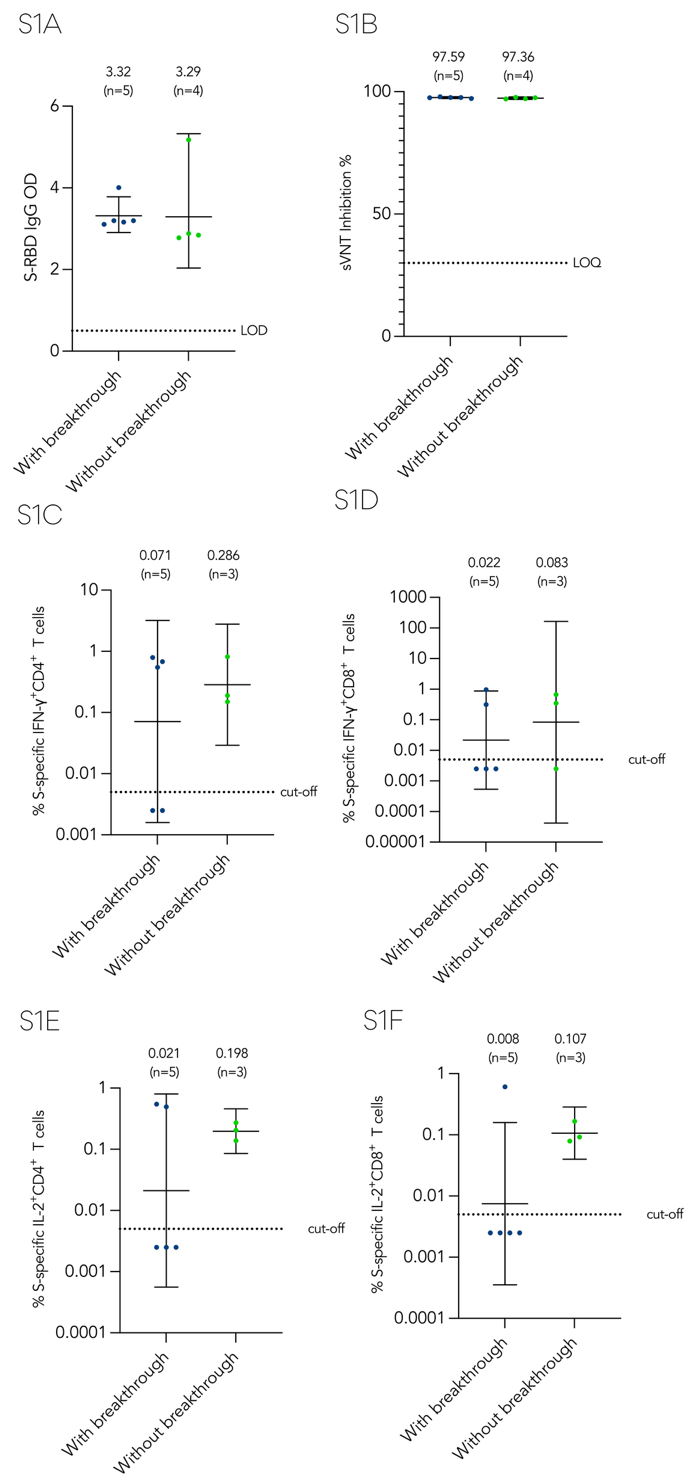


**Supplementary Figure 1. Humoral and cellular immunogenicity of patients with breakthrough infections at 1-month post-dose 4.**

WT S-RBD IgG ELISA optical density (OD) (A), WT sVNT inhibition % level (B)

and WT S-specific IFN-g+ IL-2+ CD4+ CD8+ (C-F) at 1-month post-dose 4 were

shown. Geometric means were indicated by centerlines and listed above each column with number of samples included. Limit of detection (0.5), limit of quantification (30%) or cut-offs (0.005) were drawn as dotted lines. OD, optical density; S-RBD, spike receptor-binding domain; sVNT, surrogate virus neutralization test; IFN-g, interferon gamma; IL-2 interleukin 2; B, BNT162b2;C, CoronaVac; S, spike; WT, wild type; LOD, limit of detection; LOQ, limit of quantification.
